# Downregulation of PRPS2 inhibits tumor growth of non-small cell lung cancer by suppressing PI3K/AKT signaling

**DOI:** 10.3389/fmed.2026.1789181

**Published:** 2026-05-12

**Authors:** Qian Xu, Qing Liu, Chunlin Ke, Peifeng Hou

**Affiliations:** 1 Department of Oncology, Fujian Medical University Union Hospital, Fuzhou, Fujian, China; 2 Fujian Key Laboratory of Translational Cancer Medicine, Fuzhou, Fujian, China; 3 Fujian Medical University Stem Cell Research Institute, Fuzhou, Fujian, China; 4 Department of Radiotherapy, Cancer Center, The First Affiliated Hospital of Fujian Medical University, Fuzhou, China; 5 Key Laboratory of Radiation Biology of Fujian Higher Education Institutions, The First Affiliated Hospital, Fujian Medical University, Fuzhou, China

**Keywords:** non-small cell lung cancer, PI3K/AKT signaling, pristimerin, PRPS2, tumor growth

## Abstract

**Introduction:**

This study aims to investigate the role of phosphoribosyl pyrophosphate synthetases 2 (PRPS2) in non-small cell lung cancer (NSCLC) and its underlying molecular mechanisms.

**Methods:**

The expression patterns of PRPS2 were analyzed in databases, and its expression levels were validated by RT-qPCR. Stable cell lines with PRPS2 knockdown and overexpression were established. In addition, MTT and colony formation assays were conducted to determine cell proliferation. Western blotting and RT-qPCR assays were conducted to evaluate changes in PI3K/AKT signaling and cell cycle proteins. Furthermore, a cell line-derived xenograft animal model was utilized to confirm the roles of PRPS2 *in vivo*.

**Results:**

An elevation of PRPS2 was observed in NSCLC samples, and its overexpression promoted NSCLC cell proliferation, while PRPS2 knockdown inhibited cell proliferation. Additionally, PRPS2 overexpression promoted the PI3K/AKT signaling pathway in NSCLC cells. Moreover, PRPS2 regulated the expression of downstream genes in the PI3K/AKT pathway. Consistently, PRPS2 knockdown suppressed NSCLC tumor growth *in vivo*. In addition, PRPS2 was found downregulated by the natural compound pristimerin in NSCLC cells.

**Conclusion:**

PRPS2 promotes tumor growth of NSCLC through the regulation of the activation of the PI3K/AKT signaling pathway. Targeting PRPS2 may be a promising strategy for the therapy of NSCLC in the future.

## Introduction

Lung cancer remains a major public health challenge worldwide, significantly impacting both morbidity and mortality rates (1). Lung cancer includes non-small cell lung cancer (NSCLC) and small cell lung cancer, where NSCLC accounts for approximately 80%∼ 85%, and small cell lung cancer comprises 10%∼15% (1, 2). According to projections by the American Cancer Society, the United States is expected to witness around 234,580 new cases of lung cancer and approximately 125,070 lung cancer-related deaths in 2024 (3). Despite advancements in therapeutic approaches, the prognosis for NSCLC patients remains unfavorable, emphasizing the urgent need for a thorough comprehension of the molecular mechanisms driving its pathogenesis and progression (4).

Considerable research efforts have been directed toward unraveling the molecular landscape of NSCLC and identifying novel therapeutic targets to enhance patient outcomes in the recent years (4). Among the emerging targets of interest is phosphoribosyl pyrophosphate synthetase 2 (PRPS2), an enzyme crucial for de novo purine nucleotide biosynthesis, which also plays a pivotal role in pyrimidine and purine synthesis (5, 6). While traditionally recognized for its involvement in cellular metabolism, accumulating evidence suggests that PRPS2 exerts a multifaceted influence on cancer development and progression (7–10). Studies have implicated dysregulated PRPS2 expression in various malignancies, including NSCLC, where it has been associated with tumor aggressiveness, metastasis, and therapy resistance (7, 8). Furthermore, aberrant PRPS2 expression has been correlated with unfavorable clinical outcomes in NSCLC patients (7), highlighting its potential as a prognostic biomarker. For instance, PRPS2 has been implicated in cell proliferation and metastasis in neuroblastoma malignancy, and it plays an important role in Myc-driven cancers in both murine and human models (5).

Although PRPS2 has been implicated in NSCLC pathogenesis, its expression correlation with well-established driver oncogenes in NSCLC—such as EGFR, ALK, and KRAS—remains largely unexplored. Previous studies have suggested that PRPS2 expression may be independent of these common driver mutations, potentially offering a complementary or synergistic therapeutic target (11). Notably, in NSCLC tumors lacking canonical driver alterations, metabolic enzymes like PRPS2 may play a particularly prominent role in sustaining proliferative capacity through enhanced nucleotide biosynthesis. Therefore, understanding the relationship between PRPS2 and major driver pathways could help identify patient subsets that might benefit most from PRPS2-targeted therapies and may reveal previously unrecognized crosstalk between metabolic regulation and oncogenic signaling in NSCLC.

The phosphatidylinositol 3-kinase (PI3K)/protein kinase B (AKT) signaling pathway has emerged as a central regulator of essential cellular processes, including proliferation, growth, survival, and metabolism (12–14). Dysregulation of this pathway is a hallmark of cancer, including NSCLC, where it contributes to tumor initiation, progression, and therapeutic resistance (15, 16). Notably, an interplay between PRPS2 and the PI3K/AKT pathway has been suggested in modulating cancer pathobiology (6, 17), indicating potential synergistic effects in driving tumor development and progression. Beyond the intrinsic regulatory role of PRPS2 in tumor cells, the tumor microenvironment (TME) and its crosstalk with metabolic reprogramming also play pivotal roles in NSCLC progression. A recent study analyzing the crosstalk between cancer-associated fibroblasts (CAFs), immune cells, and metabolism revealed that high CAF infiltration in lung cancer is associated with unfavorable prognosis, enhanced M2 macrophage recruitment, and dysregulated glycerophospholipid metabolism (18). Moreover, CAF-enriched TME activates multiple oncogenic pathways such as IL6/JAK/STAT3 and TNF-α signaling, which are known to crosstalk with the PI3K/AKT pathway (18). Given that PRPS2 has been linked to M2 macrophage polarization in NSCLC (7), it is plausible that PRPS2 may participate in the CAF-immune-metabolism regulatory network to promote tumor progression, though this crosstalk requires further investigation.

While previous studies have highlighted the association of PRPS2 with tumorigenesis in various cancers, its precise role in NSCLC remains incompletely understood. Prior investigations from our research group have suggested a correlation between PRPS2 expression and chemotherapy resistance in NSCLC (7). Therefore, this study aims to delve deeper into the relationship between PRPS2 and cell proliferation in NSCLC, elucidating its underlying mechanisms.

## Materials and methods

### Clinical sample collection

In this study, patients were recruited at Fujian Medical University Union Hospital. The study was approved by Ethics Committee of Fujian Medical University Union Hospital. Prior to participation, all patients provided informed consent after thoroughly reviewing and signing the consent form. In total, we obtained 60 pairs of tissue samples, consisting of NSCLC tumor tissues and adjacent tissues (normal tissues), for subsequent analysis. The demographic and clinical characteristics of the 60 NSCLC patients were as listed in Supplementary Table 1.

### Cell line establishment, culture, and viability assessment

Cell lines including A549 and H1299 were procured from (ATCC, Manassas, VA). BEAS-2B, H1650 and SK-MES-1 cell lines were obtained from the Cell Bank, China Academy of Sciences (Shanghai, China). All cell lines were verified with STR profiling, and were routinely tested for mycoplasma contamination. All the cells were cultured in RPMI-1640 medium supplemented with 10% fetal bovine serum (FBS, Hyclone Laboratories, Logan, UT) and 1% Penicillin-Streptomycin (P/S) solution (Invitrogen, Waltham, MA).

To generate cell lines overexpressing PRPS2, cells were transfected with pLVX-PRPS2-derived lentivirus or pLVX-NC lentivirus as a negative control. Additionally, PRPS2 knockdown cell lines were established by transfecting cells with specific siRNAs targeting PRPS2 or negative control siRNAs for 48 h. The sequences of siRNA oligonucleotides were as follows: si-PRPS2#1: CTGCAAGATTGCGTCATCA; si-PRPS2#2: CCACCAAAG TGTATGCTAT. For lentiviral transduction, A549 and H1299 cells were seeded in 6-well plates at a density of 5 × 10^4^ cells per well and incubated overnight. The next day, the culture medium was replaced with fresh medium containing lentivirus (pLVX-NC or pLVX-PRPS2; shNC or shPRPS2) at a multiplicity of infection (MOI) of 10. Polybrene (8 μg/mL) was added to enhance transduction efficiency. After 24 h of incubation, the virus-containing medium was replaced with fresh complete medium. Stable transductants were selected using puromycin at a final concentration of 5 μg/mL for 7 days, and puromycin-resistant cells were expanded for subsequent experiments.

Cell viability was determined using MTT and colony formation assay, respectively. For the MTT assay, A549 and H1299 cells were first transfected with lentivirus (for overexpression or knockdown) or siRNAs (for transient knockdown). After 48 h of transfection, the cells were harvested and seeded into 96-well plates at a density of 5 × 10ł cells per well in 100 μL of complete medium. After culturing for 24, 48, or 72 h, 10 μL of MTT solution (5 mg/mL, Sigma, St. Louis, MO) was added to each well and the plates were incubated for an additional 4 h at 37°C. The formazan crystals were then dissolved in 100 μL of dimethyl sulfoxide (DMSO). The absorbance was measured at 570 nm using a microplate reader (Bio-Rad, Hercules, CA).

For the colony formation assay, cells that had undergone transfection with lentivirus or siRNAs were seeded into triplicate wells within 6-well plates (1 × 10^3^ cells per well). After that, these cells were then cultured in complete medium for a duration of 2 weeks. Following this period, the colonies were immobilized using 4% paraformaldehyde and subjected to staining with Wright Giemsa stain for 10 min prior to quantification.

For the pristimerin (PTM, #S9404, Selleck chemicals) treatment, A549 and H1299 cells were incubated with increasing concentrations of PTM for 24 h, then subjected to biochemical assays.

### RT-qPCR assays

Total RNAs were extracted by using TRIzol reagent (Thermo Fisher, Waltham, MA). An amount of 500 ng of RNA underwent reverse transcription into cDNA using the RT-PCR reagents (TaKaRa, Japan). Next, real-time PCR was performed using a reaction mixture containing Master Mix and the RNA reverse transcription product as the template. 2-^ΔΔ^Ct method was used for the gene expression quantification. ^Δ^Ct indicates the difference in cycle threshold values. GAPDH served as the internal control for normalization.

The following primer sequences were used: PRPS2: Forward primer, 5′- AGCTCGCATCAGGACCTGT-3′; Reverse primer, 5′-ACGCTTTCACCAATCTCCACG-3′. Cyclin D1: Forward primer, 5′-CTCTAAGATGAAGGAGACCAT-3′; Reverse primer, 5′-TTGGAGAGGAAGTGTTCAA-3′. Bcl-2: Forward primer, 5′-GTGGATGACTGAGTACCTGAAC-3′; Reverse primer, 5′-GAGACAGCCAGGAGAAATCAA-3′. MKI67: Forward primer, 5′-TCAGCACCTGCTTGTTTGGA-3′; Reverse primer, 5′-TTGCCTCCTGCTCATGGATT-3′. GAPDH (internal control): Forward primer, 5′-CCAGGTGGTCTCCTCTGACTTC-3′; Reverse primer, 5′-GTGGTGTTGAGGGCAATG-3′.

### Western blot analysis

Primary antibodies including anti-PRPS2 (1:2,000, Abcam, Cambridge, United Kingdom), anti-PRPS1 (1:1,000, Abcam, Cambridge, United Kingdom), anti-p-PI3K p85 (Tyr458)/p55 (Tyr199) (1:1,000, CST, Massachusetts, United States), anti-PI3K p85 (1:1,000, CST, Massachusetts, United States), anti-p-AKT (Ser 473) (1:1,000, Abcam, Cambridge, United Kingdom), anti-AKT (1:2,000, Abcam, Cambridge, United Kingdom), anti-p-mTOR (Ser2448) (1:1,000, CST, Massachusetts, USA), anti-mTOR (1: 1000, CST, Massachusetts, USA), anti-Cyclin D1 (1: 1000, CST, Massachusetts, United States), anti-Bcl-2 (1: 1,000, CST, Massachusetts, United States) and anti-GAPDH (1:3,000, Abcam, Cambridge, United Kingdom) were used. Proteins were extracted by using RIPA buffer plus 1% (v/v) protease inhibitor cocktail. The samples were collected and homogenized in cold RIPA buffer. After that, the samples were undergoing lysis for another 30 min. The protein concentration of each sample was measured using the bicinchoninic acid assays. Next, protein samples were separated by 10% SDS-PAGE gels. Then, the gels were transferred onto polyvinylidene fluoride membranes. After blocking with 5% BSA, the membranes were incubated with primary antibodies. After washing, the membranes were incubated with the secondary antibodies. The intensities of protein bands were then quantified using the ImageJ Software.

### Establishment of cell line-derived xenograft animal model

All experiments involving BALB/c nude male mice were approved by the Animal Care and Use Committee of Fujian Medical University Union Hospital (#2023-Y-0808), and was performed in strict accordance with the NIH guidelines for the care and use of laboratory animals (8th edition, NIH). For the establishment of the cell line-derived xenograft animal model, A549 cells were infected with sh-PRPS2 (CCACCAAAGTGTATGCTAT) lentivirus or empty lentivirus vector shNC. Thereafter, stable cell lines were selected by 5 μg/mL puromycin for 1 week. Next, we subcutaneously injected the cells (5 × 10^6^ in 100 μL PBS) into the right flanks of the mice. Each group has 6 mice. Tumor volume was checked every 3 days, and calculated using the formula: A × B^2^ × 0.5 (where A represents the longest diameter of the tumor and B represents the shortest diameter of the tumor, both in millimeters, and the resulting volume is in mmł). Tumor burden endpoint: tumor growth was monitored until the tumor volume reached ≤ 1,000 mmł (or up to day 21, whichever occurred first). At the end of the animal experiment, mice were euthanized, and tumors were excised for weighing.

### Statistical analysis

The data were presented as mean ± standard deviation (SD). In this study, GraphPad Prism software was applied for data analysis (GraphPad Software Inc., United States). Unpaired Student’s *t*-test was conducted for comparisons between two groups. In addition, one-way ANOVA with Tukey’s *post-hoc* tests was used for multiple comparisons. A *p*-value that was less than 0.05 was used to show statistical significance.

## Results

### Elevated PRPS2 promoted NSCLC cell proliferation

**FIGURE 1:**
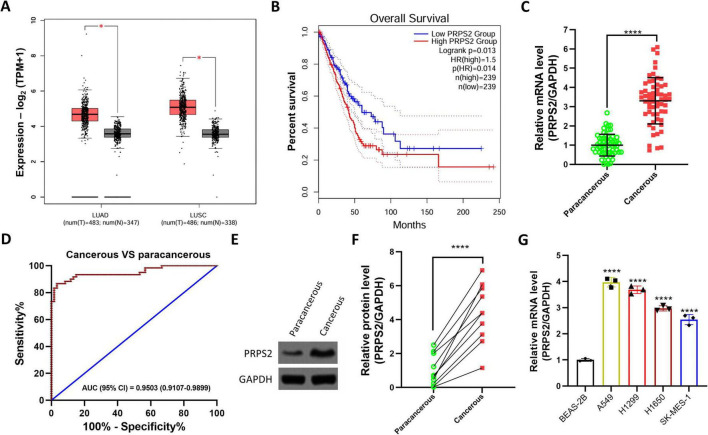
Elevated PRPS2 is predicted as a poor index for NSCLC. **(A)** The public tumor database GEPIA 2 (http://gepia2.cancer-pku.cn) was used to analyze the expression of PRPS2 in LUAD (lung adenocarcinoma) and LUSC (lung squamous carcinoma). **(B)** The overall survival of LUAD patients with low or high PRPS2 was retrieved from GEPIA 2(http://gepia2.cancer-pku.cn/#survival). **(C)** RT-qPCR was carried out to analyze the mRNA levels of PRPS2 in 60 pairs of paracancerous and cancerous NSCLC tissues. The data was shown as Mean ± standard deviation. **(D)** ROC curve was used to investigate the value of PRPS2 in the diagnosis of NSCLC. **(E,F)** Ten pairs of tumor tissues were randomly selected and used for western blot analysis to detect the protein expression level of PRPS2 (representative image was shown in **E**), and optical density analysis was also performed (*n* = 10). **(G)** BEAS-2B, A549, H1299, H1650 and SK-MES-1 cell lines were prepared for RT-qPCR to assess the mRNA levels of PRPS2 (*n* = 3). GAPDH was used an internal control. The data was shown as Mean ± standard deviation. *p < 0.05, ****p < 0.0001.

First, we conducted an analysis using the publicly available cancer database GEPIA to examine the expression pattern of PRPS2 in NSCLC, specifically focusing on lung adenocarcinoma (LUAD) and lung squamous cell carcinoma (LUSC). Our investigation revealed a notable upregulation of PRPS2 in LUAD and LUSC (Figure 1A). In addition, the database also indicated that NSCLC patients with high PRPS2 had a shorter overall survival (Figure 1B); however, this analysis is univariate and exploratory in nature, and multivariate analysis was not performed. Subsequently, we obtained and analyzed 60 pairs of tumor and adjacent tissues using RT-qPCR to validate the observed expression patterns. Our results confirmed a significant overexpression of PRPS2 in NSCLC compared to adjacent normal tissues (Figure 1C). ROC curve was also used to investigate the value of PRPS2 in the diagnosis of NSCLC (Figure 1D). In addition, the protein-level validation of PRPS2 was also performed in patient tumor tissues (Figures 1E,F). Then, a normal human lung epithelial cell and four NSCLC cell lines were collected and prepared for RT-qPCR. As shown in Figure 1G, PRPS2 was significantly upregulated in NSCLC cell lines compared with the normal cell line.

**FIGURE 2:**
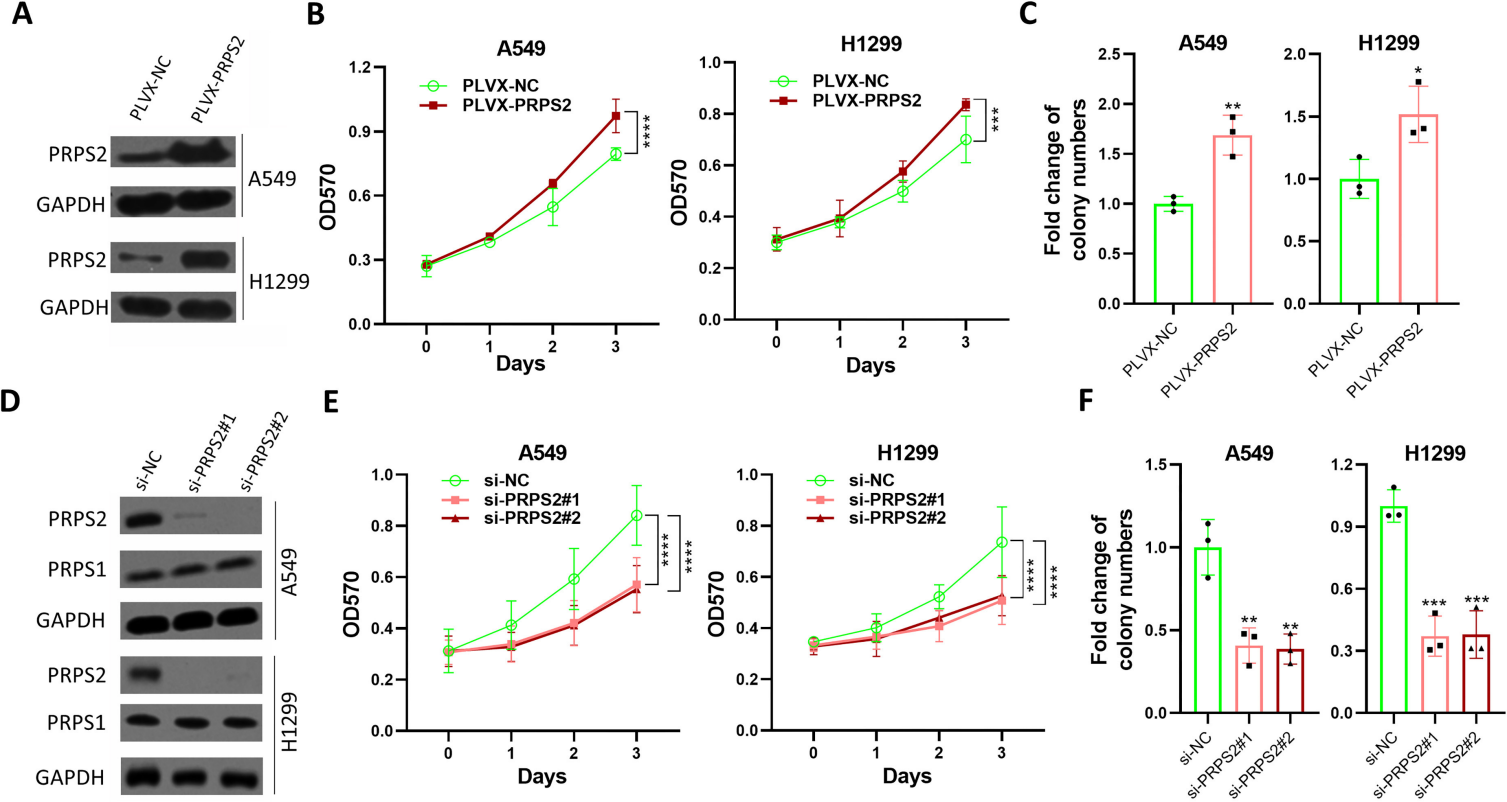
PRPS2 promotes cell proliferation of NSCLC. **(A)** A549 and H1299 cells stably infected with PLVX-NC or PLVX-PRPS2-derived lentivirus were lysed for western blot to analyze the expression of PRPS2. GAPDH was used as a loading control. **(B)** Indicated cells were prepared for MTT assay to analyze the cell proliferation of NSCLC (*n* = 5). **(C)** Indicated cells were also prepared for colony formation assay (*n* = 3). **(D)** A549 and H1299 cells were transfected with indicated siRNAs for 48 h, and then cells were lysed for western blot to analyze the expression of PRPS2 and PRPS1. GAPDH was used as a loading control. **(E)** A549 and H1299 cells transfected with indicated siRNAs were prepared for MTT assay to analyze the cell proliferation of NSCLC (*n* = 5). **(F)** Indicated cells transfected with indicated siRNAs were also prepared for colony formation assay (*n* = 3). NSCLC, non-small cell lung cancer. All the data was shown as Mean ± standard deviation. *p < 0.05, **p < 0.01, ***p < 0.001, ****p < 0.0001.

To further elucidate the functional implications of PRPS2 in NSCLC, we successfully overexpressed PRPS2 in NSCLC cell lines (Figure 2A). Notably, our findings demonstrated a considerable enhancement in cell growth among cells overexpressing PRPS2 (Figure 2B). Furthermore, consistent with these observations, the colony formation assay revealed an increased number of colonies in cells with elevated PRPS2 expression (Figure 2C). Furthermore, we successfully employed siRNA to silence PRPS2 expression in cells (Figure 2D). Our results demonstrated that knockdown of PRPS2 dramatically suppressed the growth of cells compared to those transfected with si-NC (Figure 2E and Supplementary Figures 1A,B). Consistently, the colony formation assay revealed a reduced number of colonies in cells with decreased PRPS2 expression (Figure 2F and Supplementary Figures 1C,D). Knockdown of PRPS2 by siRNAs also significantly induced cell cycle arrest at G2/M phase, induced cell apoptosis, and suppressed cell migration in NSCLC cells (Supplementary Figure 2). Thus, we have provided evidence from both positive and negative aspects, suggesting a positive correlation between PRPS2 expression and NSCLC cell proliferation.

### PRPS2 activated PI3K/AKT signaling in NSCLC cell lines

**FIGURE 3:**
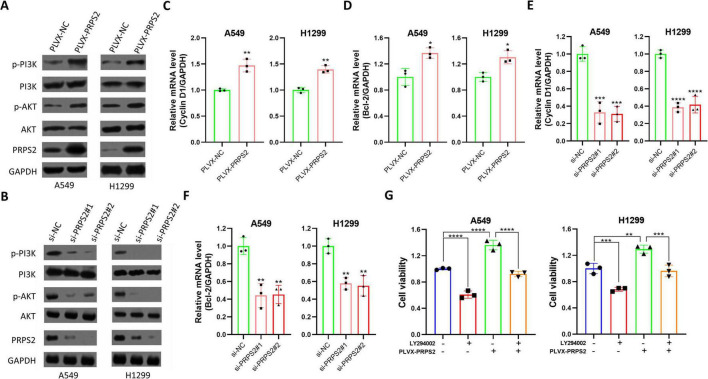
PRPS2 activates PI3K/AKT signaling in NSCLC cells. **(A)** A549 and H1299 cells stably infected with PLVX-NC or PLVX-PRPS2-derived lentivirus were lysed for western blot to analyze the expression of p-PI3K p85 (Tyr458)/p55 (Tyr199), PI3K p85, p-AKT (Ser 473), AKT and PRPS2. GAPDH was used as a loading control. **(B)** A549 and H1299 cells were transfected with indicated siRNAs for 48 h, and then cells were lysed for western blot to analyze the expression of p-PI3K p85 (Tyr 458)/p55 (Tyr199), PI3K p85, p-AKT (Ser 473), AKT and PRPS2. GAPDH was used as a loading control. **(C,D)** A549 and H1299 cells stably infected with PLVX-NC or PLVX-PRPS2-derived lentivirus were prepared for RT-qPCR to analyze the mRNA level of Cyclin D1 **(C)** and Bcl-2 **(D)**. GAPDH was used as an internal control. **(E,F)** A549 and H1299 cells were transfected with indicated siRNAs for 48 h, and then cells were prepared for RT-qPCR to analyze the mRNA level of Cyclin D1 **(E)** and Bcl-2 **(F)**. GAPDH was used as an internal control. **(G)** A549 and H1299 cells were infected with shPRPS2-derived lentivirus or incubated with 10 μM LY294002 for 24 h, followed by MTT assay to analyze the cell viability of NSCLC. The data was shown as Mean ± standard deviation (*n* = 3 for in vitro experiments). *p < 0.05, **p < 0.01, ***p < 0.001, ****p < 0.0001.

To investigate the mechanisms by which PRPS2 promotes cell proliferation in NSCLC, we investigated the protein expression of both PI3K and AKT, which is associated with cell proliferation. Our data showed that PRPS2 overexpressing significantly enhanced the phosphorylation of both PI3K and AKT (Figure 3A). In addition, the phosphorylation levels of the key downstream molecule mTOR in the pathway were also enhanced after the overexpression of PRPS2 (Supplementary Figure 3A). Conversely, silencing of PRPS2 resulted in a marked inhibition of PI3K and AKT protein phosphorylation (Figure 3B). These findings further suggested that PRPS2 promoted NSCLC cell proliferation by the regulation of the PI3K/AKT signaling pathway. We also assessed the levels of downstream target genes associated with cell proliferation in the PI3K/AKT signaling pathway. We found that PRPS2 overexpressing significantly increased the levels of Cyclin D1 and Bcl-2 in A549 and H1299 (Figures 3C,D and Supplementary Figure 3B). Conversely, silencing of PRPS2 led to a reduction in the levels of these genes (Cyclin D1 and Bcl-2, Figures 3E,F). Moreover, PRPS2-induced NSCLC cell survival could be reversed by the PI3K inhibitor LY294002 (Figure 3G). These results further underscore the close association between PRPS2 and NSCLC cell proliferation.

### PRPS2 knockdown inhibited NSCLC tumor growth *in vivo*

**FIGURE 4:**
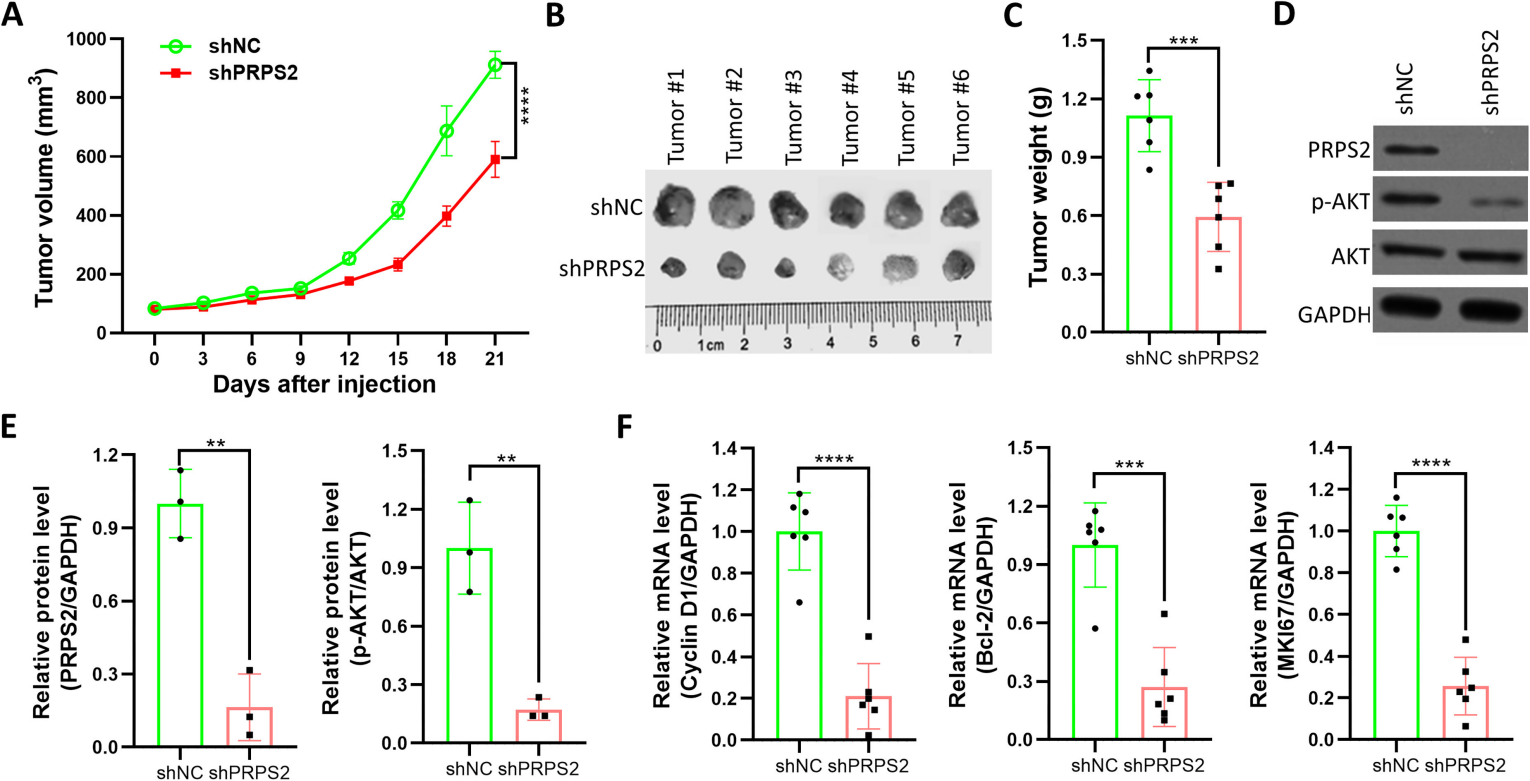
PRPS2 knockdown inhibits tumor growth of NSCLC *in vivo*. **(A)** A549 cells stably infected with shNC or shPRPS2-derived lentivirus were subcutaneously injected into the right flanks of nude mice (*N* = 6 per group). Tumor volumes were monitored every 3 days for continuously 3 weeks. Tumor growth curves were drawn as indicated. **(B,C)** On Day 21, mice were sacrificed and tumors were excised for taken photos **(B)**, and weighing **(C)**. **(D,E)** Tumors (from 3 randomly selected mice per group, *n* = 3) were homogenized for western blot analysis of PRPS2, p-AKT, AKT, and GAPDH **(D)**, and the indicated optical density was also analyzed **(E)**. **(F)** Tumors were also prepared for RT-qPCR to analyze the mRNA level of Cyclin D1, Bcl-2 and MKI67. GAPDH was used as an internal control. The data was shown as Mean ± standard deviation. **p < 0.01, ***p < 0.001, ****p < 0.0001.

Subsequently, to establish a xenograft tumor model, we subcutaneously injected stable PRPS2 shRNA transfected A549 cells. Our data revealed that PRPS2 knockdown significantly suppressed tumor volume and weight (Figures 4A–C). Furthermore, biochemical analysis validated the downregulation of PRPS2 within the tumor (Figures 4D,E), along with a decrease in the activation levels of AKT (Figures 4D,E). In addition, the expression of the downstream target genes of PI3K/AKT signaling, including Cyclin D1 and Bcl-2, were significantly downregulated in shPRPS2 group (Figure 4F). And the expression of the proliferative gene MKI67 was also downregulated in shPRPS2 group (Figure 4F). These results further suggested that PRPS2 played a pro-oncogenic role in NSCLC by modulating the PI3K/AKT signaling pathway.

### Pristimerin downregulated the expression of PRPS2 in NSCLC cells

**FIGURE 5:**
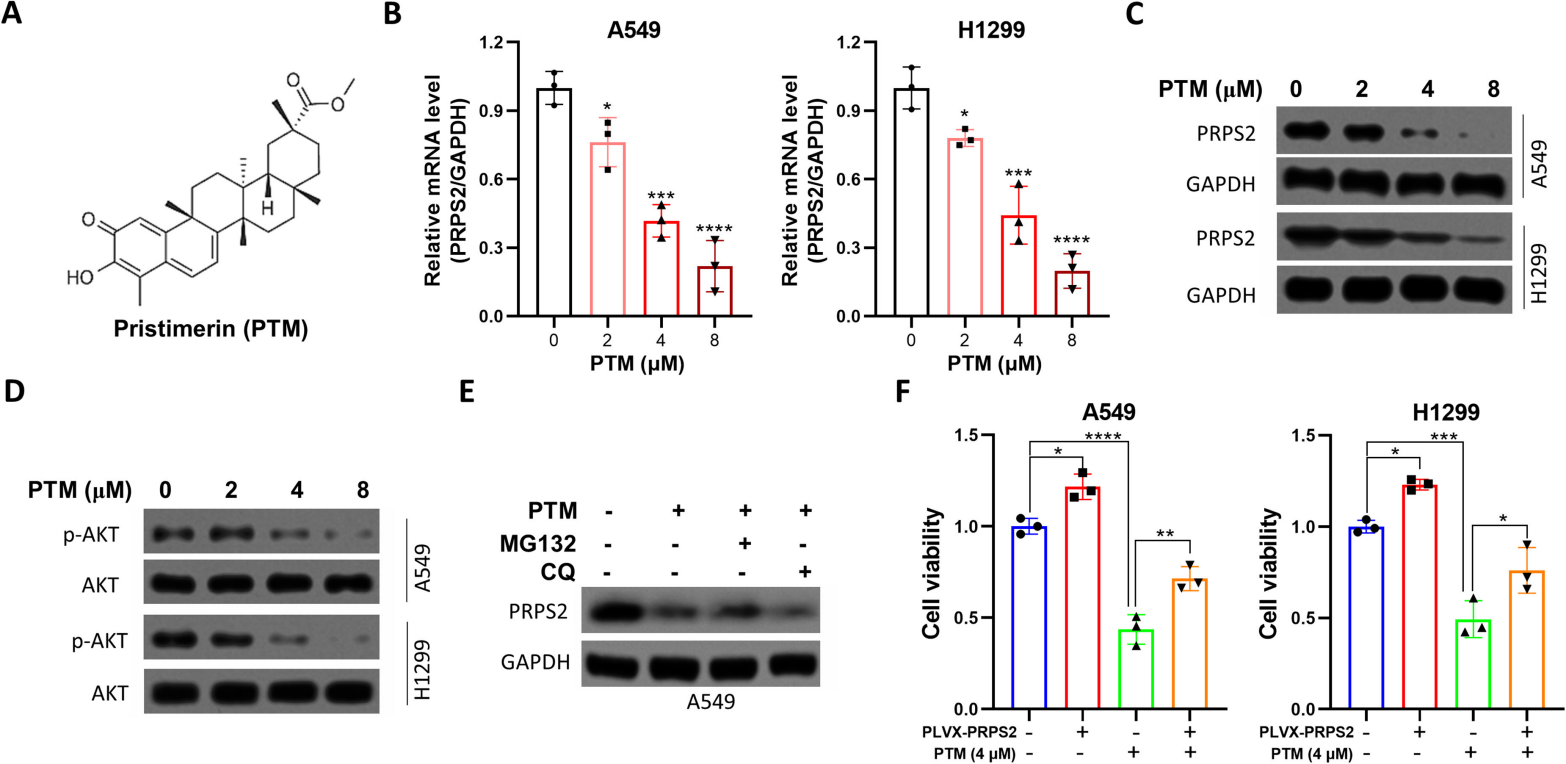
PRPS2 is downregulated by pristimerin treatment in NSCLC cells. **(A)** The chemical structure of pristimerin (PTM). **(B)** A549 and H1299 cells were incubated with increasing concentrations of PTM for 24 h, followed by RT-qPCR to analyze the mRNA levels of PRPS2. GAPDH was used as an internal control. **(C,D)** Above cells were also lysed for western blot to analyze the expression of PRPS2, p-AKT and AKT. GAPDH was used as a loading control. **(E)** A549 cells were incubated with 4 μM PTM, 10 μM MG132 or 10 μM chloroquine (CQ) for 24 h, followed by western blot to analyze the expression of PRPS2. GAPDH was used as a loading control. **(F)** A549 and H1299 cells were infected with shNC or shPRPS2-derived lentivirus for 24 h, and then cells were incubated with 4 μM PTM for 24 h, followed by MTT assay to analyze the cell viability of NSCLC. The data was shown as Mean ± standard deviation (*n* = 3 for *in vitro* experiments). *p < 0.05, **p < 0.01, ***p < 0.001, ****p < 0.0001.

Finally, the NSCLC cells were incubated with PTM, and the cell viability was significantly inhibited by PTM (Supplementary Figure 4). In addition, we also found that the mRNA levels of PRPS2 could be significantly downregulated by the treatment of PTM in NSCLC cells (Figures 5A,B). To further confirm the downregulation effects of PTM on PRPS2, NSCLC cells were incubated with indicated concentrations of PTM for 24 h, and then cells were lysed for Western blot. As shown in Figures 5C,D, PTM dramatically inhibited the protein levels of PRPS2 in both of A549 and H1299 cells; additionally, the activation of AKT was also suppressed by the treatment of PTM. The following experimental results demonstrated that concurrent treatment with the proteasome inhibitor MG132 or lysosome inhibitor chloroquine did not abrogate the PTM-induced downregulation of PRPS2 protein expression, which further confirmed that PTM suppressed the transcriptional expression of PRPS2 in NSCLC cells (Figure 5E). Finally, we found that overexpression of PRPS2 could partly abolish the cytotoxicity of PTM in NSCLC cells (Figure 5F). Collectively, PTM may exert its anti-tumor activity by downregulating PRPS2/PI3K/AKT axis in NSCLC cells.

## Discussion

In the present study, we reported that elevated PRPS2 levels promoted NSCLC cell proliferation, as evidenced by an increased proliferation rates and colony formation abilities in cells overexpressing PRPS2. Conversely, PRPS2 knockdown inhibited NSCLC cell proliferation, as indicated by a reduction in cell proliferation with reduced PRPS2 expression. Moreover, our findings suggested that PRPS2 regulated NSCLC cell proliferation by the activation of the PI3K/AKT signaling pathway, as demonstrated by enhanced phosphorylation of PI3K and AKT proteins upon PRPS2 overexpression and decreased phosphorylation levels upon PRPS2 knockdown. Additionally, PRPS2 affected the expressions of downstream genes involved in cell proliferation, such as Cyclin D1 and Bcl-2. Furthermore, *in vivo* studies confirmed that PRPS2 knockdown suppressed NSCLC tumor growth, highlighting that PRPS2 may serve as a therapeutic target for NSCLC treatment. We acknowledge that the survival association inferred from the GEPIA database is exploratory and univariate, and our clinical samples were not linked to survival outcomes. Therefore, the prognostic value of PRPS2 in NSCLC requires further validation in prospective cohorts with multivariate analysis.

Initially, to analyze the expression of PRPS2 in NSCLC, we utilized the publicly available cancer database, GEPIA (a server for normal and cancer gene profiling) (19, 20). The main subtypes of NSCLC are LUAD and LUSC (21, 22). In our analysis, we revealed a significant upregulation of PRPS2 in both LUAD and LUSC. Subsequently, we collected 60 pairs of tumor and adjacent normal tissues for validation using RT-qPCR, confirming significant overexpression of PRPS2 in NSCLC. The oncogenic role of PRPS2 in LUSC is consistent with recent findings on other LUSC-specific drivers: for instance, FAM83A, another gene highly upregulated in LUSC tissues and cell lines, promotes LUSC progression by activating the ERK pathway to induce epithelial-mesenchymal transition (EMT) and inhibit apoptosis (23). Similar to PRPS2, FAM83A regulates key cell cycle and survival-related molecules such as Cyclin D1 and Bcl-2 (23), highlighting that dysregulation of cell proliferation and apoptotic pathways is a common oncogenic mechanism in LUSC. However, distinct from FAM83A’s dependence on the ERK pathway (23), our study demonstrates that PRPS2 exerts its pro-tumor effects primarily through activating the PI3K/AKT signaling axis, suggesting that multiple parallel oncogenic pathways may coexist in LUSC to drive tumor progression.

To explore the roles of PRPS2 in NSCLC cells, we overexpressed PRPS2 in NSCLC cells and observed a significant enhancement in cell proliferation and the formation of cell colony. Conversely, silencing PRPS2 using siRNA technology significantly inhibited NSCLC cell proliferation and colony formation. Thus, we provided evidence confirming the close association between PRPS2 and NSCLC cell proliferation. In addition, our findings are consistent to previous studies, where PRPS2 knockdown inhibited tumor growth in prostate cancer cells (*in vitro* and *in vivo*) (10). Another study suggested PRPS2 serving as a cancer promoter in colon cancers (8).

To further elucidate the underlying mechanisms of PRPS2-mediated promotion of cell proliferation in NSCLC, we determined the PI3K/AKT signaling pathway, which is also known to be associated with cell proliferation (24). Abnormal PI3K/AKT signaling activation is widely found in many types of cancers and is associated with cell proliferation, survival, and cell cycle (25, 26). Our data demonstrated that overexpression of PRPS2 significantly promoted the phosphorylation of PI3K and AKT proteins, while silencing PRPS2 significantly inhibited their phosphorylation. In previous studies, Qiao and colleagues reported that PRPS2 promotes prostate cancer by the regulation of cell apoptosis and cell cycle (10). We found that an elevated PRPS2 promotes cisplatin resistance in NSCLC and type 2 macrophage polarization (7). In this study, our findings further suggested that PRPS2 promoted NSCLC cell proliferation by the modulation of the activation of the PI3K/AKT signaling pathway. Although this study reports that PRPS2 can significantly promote the phosphorylation of PI3K and its downstream molecules AKT/mTOR, we have not detected an interaction between PRPS2 and PI3K subunits using Co-IP experiments (data not shown). Therefore, we speculate that this regulation may depend on indirect mechanisms, such as metabolite-mediated regulation, transduction via upstream receptor adaptor proteins, or transient interactions triggered by specific stimulation conditions. In our future work, we will further explore the specific molecular pathways by which PRPS2 regulates PI3K through conditional stimulation experiments, metabolomic correlation analysis, or high-sensitivity interaction detection technologies.

Furthermore, we assessed the levels of downstream target genes (Cyclin D1 and Bcl-2) closely associated with cell proliferation in the PI3K/AKT signaling pathway (27, 28). We found that PRPS2 overexpressing significantly increased the levels of Cyclin D1 and Bcl-2, while silencing PRPS2 led to a reduction in their expression levels. These results further underscored the close association between PRPS2 and NSCLC cell proliferation. These findings are consistent with previous studies, in which PRPS2 promotes prostate cancer by the regulation of cell cycle proteins including Bcl-2 and CyclinD1 (10).

We established a CDX model by subcutaneously injecting stable PRPS2 shRNA transfected A549 cells into nude mice. CDX model is widely used for the preclinical drug development and biomedical research (29, 30). In our analysis, we revealed that PRPS2 knockdown significantly inhibited tumor growth rate. Biochemical analysis further confirmed downregulation of PRPS2 within the tumor, accompanied by decreased activation levels of AKT. These findings suggested that PRPS2 played a pro-oncogenic role in NSCLC by modulating the PI3K/AKT signaling pathway.

Our findings reveal that PTM significantly downregulates the transcriptional expression of PRPS2 in NSCLC cell lines. This downregulation was accompanied by suppression of AKT phosphorylation, suggesting that PTM may exert its cytotoxic effects via inhibition of the PRPS2/PI3K/AKT signaling axis. Notably, overexpression of PRPS2 partially reversed the cytotoxic effects of PTM, highlighting the functional relevance of PRPS2 in PTM-mediated anti-tumor activity.

PRPS2 is a key enzyme in de novo nucleotide biosynthesis. Recent studies have demonstrated that PRPS2 plays an oncogenic role in various cancers, including lung, colorectal, and prostate cancers. For instance, PRPS2 was overexpressed in NSCLC tissues (7). Similarly, PRPS2 was significantly upregulated in prostate adenocarcinoma tissues (10). Our findings are consistent with these reports and suggest that PTM’s anti-cancer efficacy in NSCLC may, at least in part, be due to PRPS2 inhibition. Although our study did not directly compare PTM with established PI3K/AKT inhibitors such as alpelisib or cabozantinib, PTM may offer distinct advantages. Unlike direct PI3K/AKT inhibitors, PTM suppresses this pathway through downregulation of PRPS2, a metabolic enzyme upstream of nucleotide biosynthesis. This unique mechanism, combined with PTM’s reported multi-targeting effects [e.g., ROS induction (31)], suggests potential efficacy in tumors resistant to conventional PI3K/AKT inhibitors. Future comparative and combination studies are warranted.

The PI3K/AKT pathway is a well-established pro-survival and pro-proliferative signaling axis frequently hyperactivated in lung cancer (13). Our observation that PTM decreases AKT phosphorylation aligns with previous reports showing that natural compounds targeting this pathway can induce apoptosis and suppress tumor progression. For example, PTM has been shown to inhibit AKT activation in breast and pancreatic cancer models, contributing to its anti-proliferative and pro-apoptotic effects (32, 33). In our study, this inhibitory effect on AKT was observed in conjunction with PRPS2 suppression, suggesting a mechanistic link between these two events.

Moreover, the partial reversal of PTM-induced cytotoxicity following PRPS2 overexpression supports the hypothesis that PRPS2 plays a central role in regulating NSCLC cell survival. However, the incomplete rescue also implies that PTM likely affects additional oncogenic pathways beyond PRPS2/PI3K/AKT. Indeed, PTM has been reported to induce reactive oxygen species (ROS) generation, and activate caspase-dependent apoptosis (31). Thus, its multi-targeted mechanism may offer therapeutic advantages by reducing the likelihood of resistance development.

Importantly, while PRPS2 has not been widely explored as a therapeutic target, our findings support its candidacy as a vulnerability in NSCLC. Inhibiting metabolic pathways like nucleotide biosynthesis is an emerging strategy in oncology, especially in tumors that exhibit high proliferative rates and metabolic demands (34). Our study contributes to this growing area by identifying a natural compound that modulates this pathway.

Our study provides novel insights into the anti-tumor mechanism of PTM in NSCLC, demonstrating that it suppresses PRPS2 expression and downstream PI3K/AKT signaling. These results are consistent with previous findings regarding the oncogenic role of PRPS2 and the therapeutic relevance of the PI3K/AKT pathway in lung cancer. However, in terms of the xenograft model construction, due to the long- term nature of animal experiments and limited research funding, we only used the A549 cell line to establish the xenograft model. Future *in vivo* studies and clinical validation will be essential to establish PTM or its analogs as potential therapeutic agents targeting the PRPS2/PI3K/AKT axis in NSCLC.

## Conclusion

Our study revealed that elevated PRPS2 levels promoted NSCLC cell proliferation, while PRPS2 knockdown inhibited proliferation. This effect is mediated through the activation of the PI3K/AKT signaling pathway and regulation of downstream genes including Cyclin D1 and Bcl-2. Our *in vivo* experiments confirmed that PRPS2 knockdown suppressed NSCLC tumor growth, suggesting its potential as a therapeutic target. However, the prognostic significance of PRPS2 requires further validation in larger cohorts with multivariate analysis and survival data. Additionally, PTM could downregulate the expression of PRPS2, highlighting the potential of targeting PRPS2 for the NSCLC treatment.

## Data Availability

The original contributions presented in this study are included in the article/Supplementary material, further inquiries can be directed to the corresponding authors.
