# Supplementary material for: Downregulation of PRPS2 inhibits tumor growth of non-small cell lung cancer by suppressing PI3K/AKT signaling
Source: Front Med (Lausanne). 2026 May 12;13:1789181. doi: 10.3389/fmed.2026.1789181 (PMC13201483; doi:10.3389/fmed.2026.1789181)
Supplement: Supplementary file 1 [file Data_Sheet_1.docx]

**Supplementary materials**

**Supplementary methods**

*Cell cycle analysis*

A549 and H1299 cells were transfected with the indicated siRNAs and cultured for 72 hours. Subsequently, cells were harvested, washed with cold PBS, and fixed in 75% ethanol at 4°C overnight. Fixed cells were centrifuged, resuspended, and incubated with RNase at 37°C for 30 minutes, followed by propidium iodide (PI) staining. After removing residual RNase and PI, cells were resuspended in 500 µL of 1×binding buffer. The DNA content and cell cycle distribution were analyzed by flow cytometry.

*Cell apoptosis analysis*

A549 and H1299 cells were transfected with the indicated siRNAs and cultured for 72 hours. Cells were then harvested, washed three times with ice-cold PBS, centrifuged, and resuspended in 500 µL of 1×binding buffer. Finally, cells were stained with Annexin V-FITC and PI (Beyotime, Beijing, China) for 15 minutes at 37°C in the dark, and cell apoptosis was analyzed by flow cytometry.

*Cell migration assay*

To assess cell migration, approximately 40,000 cells suspended in 300 μL of serum-free RPMI 1640 medium were seeded into the upper compartment of Transwell chambers with an 8 μm pore size membrane. The lower compartment was filled with 800 μL of RPMI 1640 medium supplemented with 10% fetal bovine serum (FBS) as a chemoattractant. After 24 hours of incubation, cells that had migrated to the lower surface of the membrane were fixed with 4% paraformaldehyde, stained with 0.5% crystal violet, and counted under a light microscope.

**Supplementary figures**

**Figure S1. Knockdown of PRPS2 inhibits cell growth of NSCLC. A & B.** H1650 (A) and SK-MES-1 (B) cells transfected with indicated siRNAs were prepared for MTT assay to analyze the cell growth of NSCLC. The data was shown as Mean ± standard deviation (n=5). **C & D.** Indicated cells transfected with indicated siRNAs were also prepared for colony formation assay. NSCLC, non-small cell lung cancer. The data was shown as Mean ± standard deviation (n=3). ^*^*p*<0.01, ^**^*p*<0.01, ^****^*p*<0.0001.

**Figure S2. PRPS2 knockdown inhibits cell cycle progression, induces cell apoptosis and suppresses cell migration of NSCLC. A & B.** A549 and H1299 cells were transfected with the indicated siRNAs and cultured for 72 hours, followed by cell cycle analysis. **C & D.** A549 and H1299 cells were transfected with the indicated siRNAs and cultured for 72 hours, followed by cell apoptosis analysis. **E & F.** The cells transfected with indicated siRNAs were used for migration assay. The data was shown as Mean ± standard deviation (n=3). ^*^*p*<0.01, ^**^*p*<0.01, ^***^*p*<0.001, ^****^*p*<0.0001.


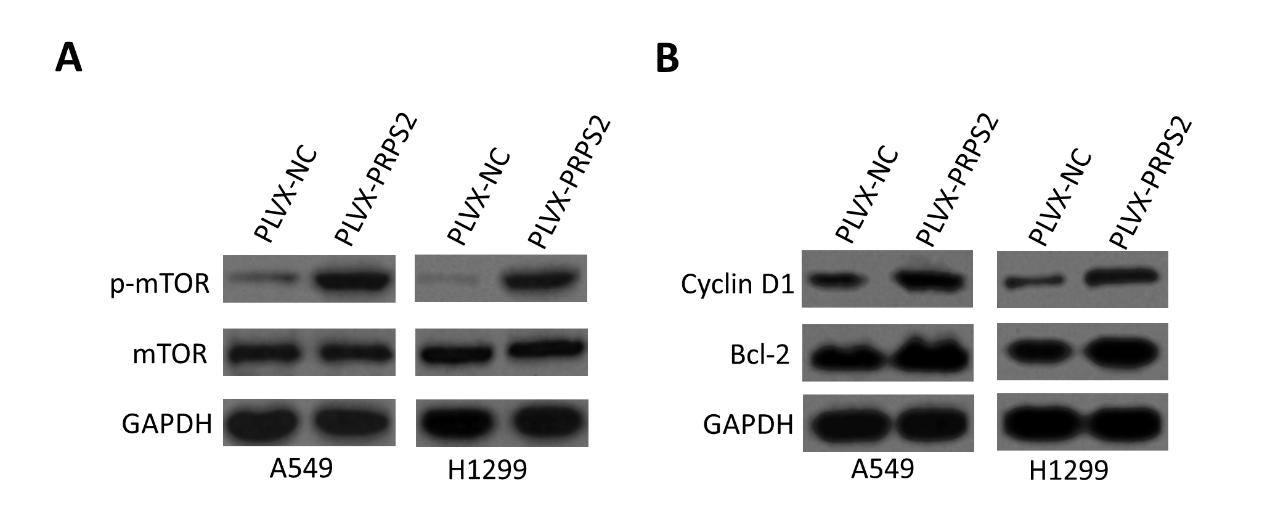


**Figure S3. PRPS2 activates the phosphorylation of mTOR in NSCLC cells. A.** A549 and H1299 cells stably infected with PLVX-NC or PLVX-PRPS2-derived lentivirus were lysed for western blot to analyze the expression of p-mTOR (Ser2448), mTOR and GAPDH. **B.** Above cells were also lysed for western blot to analyze the expression of Cyclin D1, Bcl-2 and GAPDH.

**Figure S4. PTM inhibits cell viability of NSCLC. A & B.** A549 (A) and H1299 (B) cells were incubated with indicated increasing concentrations of pristimerin (PTM) for 24 hours, followed by MTT assay. The data was shown as Mean ± standard deviation (n=3). ^**^*p*<0.01, ^****^*p*<0.0001.

**Supplementary tables:**

**Table S1. Demographic and clinical characteristics of 60 NSCLC patients.**

| **Parameter** | **N (%)** |
| --- | --- |
| **Gender** |  |
| Male | 36 (60.0%) |
| Female | 24 (40.0%) |
| **Age (years)** | 28 (46.7%) |
| ≤ 60 | 32 (53.3%) |
| > 60 |  |
| **Smoke status** |  |
| Yes | 37 (61.7%) |
| No | 23 (38.3%) |
| **Clinical stage** |  |
| I-II | 26 (43.3%) |
| III-IV | 34 (56.7%) |
| **Chemotherapy** |  |
| Yes | 21 (35%) |
| No | 39 (65%) |
| **Radiotherapy** |  |
| Yes | 10 (16.7%) |
| No | 50 (83.3%) |

NSCLC, non-small cell lung cancer. N, number.
